# Production of a Human Histamine Receptor for NMR Spectroscopy in Aqueous Solutions

**DOI:** 10.3390/biom11050632

**Published:** 2021-04-24

**Authors:** Emma Mulry, Arka Prabha Ray, Matthew T. Eddy

**Affiliations:** Department of Chemistry, University of Florida, Gainesville, FL 32611, USA; emmamulry@ufl.edu (E.M.); ray.arkaprab@chem.ufl.edu (A.P.R.)

**Keywords:** GPCR, histamine receptors, NMR spectroscopy, drug binding, membrane proteins

## Abstract

G protein-coupled receptors (GPCRs) bind a broad array of extracellular molecules and transmit intracellular signals that initiate physiological responses. The signal transduction functions of GPCRs are inherently related to their structural plasticity, which can be experimentally observed by spectroscopic techniques. Nuclear magnetic resonance (NMR) spectroscopy in particular is an especially advantageous method to study the dynamic behavior of GPCRs. The success of NMR studies critically relies on the production of functional GPCRs containing stable-isotope labeled probes, which remains a challenging endeavor for most human GPCRs. We report a protocol for the production of the human histamine H_1_ receptor (H_1_R) in the methylotrophic yeast *Pichia pastoris* for NMR experiments. Systematic evaluation of multiple expression parameters resulted in a ten-fold increase in the yield of expressed H_1_R over initial efforts in defined media. The expressed receptor could be purified to homogeneity and was found to respond to the addition of known H_1_R ligands. Two-dimensional transverse relaxation-optimized spectroscopy (TROSY) NMR spectra of stable-isotope labeled H_1_R show well-dispersed and resolved signals consistent with a properly folded protein, and ^19^F-NMR data register a response of the protein to differences in efficacies of bound ligands.

## 1. Introduction

G protein-coupled receptors (GPCRs) initiate physiological processes by recognizing and binding a wide range of extracellular stimuli, including hormones, neurotransmitters and numerous other small molecules. Over one third of FDA-approved drugs target human GPCRs [1], and GPCRs remain top targets for drug development in both academic and industrial labs [1]. Histamine receptors are class A GPCRs and one of the earliest GPCR targets of rational drug development to treat illness. In humans, four histamine receptor subtypes regulate vital physiological processes including gastric acid secretion, smooth muscle relaxation, the release of neurotransmitters and neutrophil distribution [2]. The human H_1_ receptor (H_1_R) subtype is expressed in several different tissue types including in the brain, vascular smooth muscle, airway, liver and in lymphocytes [3]. Activation of H_1_R elicits physiological processes that include modulation of circadian rhythms [4], responses to allergens, and proinflammatory cytokine production [5]. H_1_R stimulation has also been implicated in inflammation-associated tumorigenesis [6]. H_1_R activation by endogenous histamine is blocked by widely recognized drugs known as antihistamines to prevent or reduce allergic responses. The strong interest in identifying novel H_1_R drugs has led to the development of over 40 H_1_R antagonists approved for use as therapeutics [7,8]

An H_1_R complex with the antagonist doxepin, a first-generation antihistamine drug, was among the earliest reported crystal structures of human GPCRs [9]. Molecular docking of antihistamines to the H_1_R structure provided a structural basis for the improved specificity of second-generation antihistamines, which formed interactions with amino acids unique to the H_1_R binding pocket that were not present in other GPCRs [9]. Recently a cryo-EM structure of H_1_R in complex with the Gq signaling protein was solved [10]. However, mechanisms of H_1_R activation by ligands and the formation of signaling complexes are not yet fully understood.

Nuclear magnetic resonance (NMR) spectroscopic studies of human GPCRs complement crystallographic and cryo-EM structures by providing key insights into the dynamic processes of complex formation and signal transduction [7]. A critical requirement for NMR studies of GPCRs is the production of functional, stable-isotope labeled receptors in milligram to multi-milligram quantities. The methylotrophic yeast *Pichia pastoris* offers several advantages to overcome impediments of GPCR production for NMR studies. *P. pastoris* can be cultured in defined media, allowing the incorporation of stable isotopes in the receptor by adding ^13^C, ^15^N and ^2^H stable-isotope reagents to the cell culture medium. Protein overexpression in *P. pastoris* is tightly regulated by the methanol-inducible AOX1 promoter, and *P. pastoris* can be grown to high cell densities that enable large-scale protein production. While several dozen GPCRs have been reported to be produced in *P. pastoris* [11], including adrenergic receptors [12], muscarinic receptors [13], dopamine receptors [14] and opioid receptors [15], a much smaller number of GPCRs have been successfully produced in *P. pastoris* for NMR experiments. The most studied example is the human A_2A_ adenosine receptor [16,17,18,19,20,21,22,23]. More recently, NMR data have been reported for the cannabinoid receptor type 1 [24] and orexin receptor 2 [24] proteins expressed in *P. pastoris*. 

We demonstrate the production of stable-isotope labeled human H_1_R in *P. pastoris* for NMR studies. Optimization of expression conditions resulted in an order-of-magnitude increase in expressed protein yielding 2 to 2.5 mg of purified protein per liter of cell culture. Using analytical size exclusion chromatography and fluorescence thermal shift experiments, we demonstrate that the purified receptor is monodispersed in aqueous solutions containing mixed micelles and interacts with known H_1_R ligands. NMR spectra of aqueous solutions containing H_1_R in complex with an antagonist are well dispersed, indicating a properly folded protein. Additional ^19^F NMR data of H_1_R complexes with small molecule ligands register a response to the differences in the efficacies of bound drugs, illustrating the potential for future characterization of H_1_R complexes by NMR. 

## 2. Materials and Methods 

### 2.1. Materials and Reagents

Common reagents were purchased from either MilliporeSigma (Burlington, MA, USA) or Fisher Scientific (Waltham, MA, USA). Stable-isotope labeled reagents, including (^15^NH_4_)_2_SO_4_ and D_2_O, were obtained from Cambridge Isotope Laboratories (Andover, MA, USA). n-dodecyl-b-D-maltoside (DDM) and lauryl maltose neopentyl glycol (LMNG) were purchased from Anatrace (Maumee, OH, USA), and cholesterol hemisuccinate (CHS) was purchased from MilliporeSigma (Burlington, MA, USA). The fluorescent dye 7-diethylamino-3-(4-maleimidophenyl)-4-methylcoumarin (CPM) was purchased from MilliporeSigma (Burlington, MA, USA). Electrocompetent cells of the Pichia pastoris Bg12 strain were purchased from Biogrammatics (Carlsbad, CA, USA). The PME1 restriction enzyme was purchased from New England Biolabs (Ipswitch, MA, USA). The gene encoding human H_1_R (20–229, 399–487) containing an N-terminal FLAG tag and 10 × C-terminal His tag was cloned into a pPIC9k at the BamHI and NotI restriction sites and was purchased from Genscript (Piscataway, NJ, USA). The 100 mL glass douncer and piston ‘A’ used to resuspend membranes were manufactured by Kimble Glass (Millville, NJ, USA) and purchased from MilliporeSigma (Burlington, MA, USA). Protease inhibitor cocktail was prepared with AEBSF (500 µM final concentration), E-64 (1 µM final concentration), leupeptin (1 µM final concentration), and aprotinin (150 nM final concentration), all purchased from GoldBio (St. Loius, MO, USA).

### 2.2. Transformation and Colony Screening

The H_1_R plasmid was linearized with PME1 and transformed into electrocompetent Bg12 Pichia pastoris cells. Immediately after electroporation, 1 mL of cold 1M sorbitol was added and the cells were incubated for 1 h at 30 °C and 250 rpm. Following incubation, an aliquot of the culture was plated on YPD agar plates containing 2% dextrose (*w*/*v*), 1.7% (*w*/*v*) yeast nitrogen base without amino acids, 0.5% (*w*/*v*) ammonium sulfate and 0.02% (*w*/*v*) biotin and incubated for 48 h at 30 °C.

Fifteen colonies were selected to screen for protein expression. An expression test was carried out by inoculating each colony in 4 mL BMGY media for 3 days and then transferring the cells to fresh BMMY media containing 0.5% methanol (*w*/*v*) with additional aliquots of methanol added every 12 h for 48 h total expression time. Individual transformants were then tested for protein expression using a Western blot protocol as described previously [22]. Glycerol stocks of transformants that showed higher levels of expression were prepared and stored at −80 °C for subsequent experiments.

### 2.3. Optimization of H_1_R Expression Conditions

To increase expression yields, induction conditions were varied and tested for relative amounts of expressed H_1_R by a Western blot assay. Small-scale cultures were grown in 4 mL of buffered minimal glycerol yeast (BMGY) media in culture tubes at 30 °C. After 48 h, the cultures were centrifuged and resuspended in 4 mL of buffered minimal methanol yeast (BMMY) media. Induction temperature, duration of protein induction, addition of a stabilizing ligand, and addition of DMSO prior to inducing expression were varied and Western blot analysis was done to determine highest yielding induction conditions. The most intense band was the expression conditions used for all future expression of H_1_R.

### 2.4. H_1_R Production and Purification for NMR Experiments

Several 15 mL culture tubes containing 4 mL buffered minimal glycerol media (BMGY) were inoculated with a glycerol stock of a selected transformant that was determined to have higher levels of protein expression and incubated at 30 °C and 250 rpm until reaching an optical density at 600 nm of 7 to 10. Then, 250 mL baffled flasks containing 50 mL BMGY were inoculated with the 4 mL cultures and grown at 30 °C for 2 days to an optical density of 15–20. Subsequently, 2.8 L baffled flasks containing 500 mL of BMGY were inoculated with the 50 mL cell cultures and grown at 30 °C for 3 days until reaching an optical density of 15–20. The cultures were then centrifuged at 3000× *g* for 15 min and resuspended in buffered minimal methanol media (BMMY) without methanol and returned to the incubator shaker. The temperature was reduced to 28 °C and after 4 h, doxepin was added to each culture to a final concentration of 20 µM. One hour later, methanol was added to the cultures to a final concentration of 0.5% w/v, and approximately every 12 h after additional aliquots of methanol were added until reaching a total induction time of 36 h. Twelve hours after the final methanol addition, the cells were harvested by centrifuging at 3000× *g* for 15 min and stored at −80 °C until future use. Uniformly ^15^N-labeled H_1_R was expressed by adding ^15^N ammonium sulfate to the BMGY and BMMY media.

Cell pellets containing H_1_R were resuspended in lysis buffer (50 mM sodium phosphate pH 7.0, 100 mM NaCl, 5% glycerol (*w*/*v*), and in-house-made protease inhibitor cocktail solution) and lysed with a cell disruptor (Pressure Biosciences) operating at 40,000 psi. Membranes were separated and collected by ultracentrifugation at 44,000 rpm (200,000× *g*), frozen in liquid nitrogen and stored at −80 °C for future use.

Membrane pellets were resuspended using a glass douncer in buffer (10 mM HEPES pH 7.0, 10 mM KCl, 20 mM MgCl_2_, 1 M NaCl). One hour prior to solubilizing, 20 µM doxepin, in-house-prepared protease inhibitor cocktail solution, and 2 mg/mL iodoacetamide were added to the resuspended membranes. The resuspended membranes were mixed with a buffer containing 0.5% (*w*/*v*) 2,2-didecylpropane-1,3-bis-β-D-maltopyranoside (LMNG), 0.025% cholesteryl hemisuccinate (CHS), 50 mM HEPES pH 7.0, and 500 mM NaCl for 6 h at 4 °C. The resulting aqueous solution was separated from insolubilized material using ultracentrifugation at 44,000 rpm (200,000× *g*) for 30 min. The supernatant was collected and incubated overnight at 4 °C with Co^2+^-charged affinity resin (Talon, Takara Bio USA, Mountain View, CA, USA) and 30 mM imidazole. 

The resin was then washed with 20 column volumes of buffer (25 mM HEPES pH 7.0, 500 mM NaCl, 10 mM MgCl_2_, 0.1% LMNG, 0.005% CHS, 8 mM ATP, 30 mM imidazole). Resin was then collected and washed two consecutive times with a second buffer (25 mM HEPES pH 7.0, 250 mM NaCl, 0.05% LMNG, 0.0025% CHS, 5% glycerol, 30 mM imidazole and 25 µM doxepin). The protein was then eluted with a third buffer (25 mM HEPES pH 7.0, 250 mM NaCl, 0.05% LMNG, 0.0025% CHS, 5% glycerol, 300 mM imidazole and 25 µM doxepin). A PD-10 desalting column (Cytiva, Marlborough, MA) was used to exchange the sample into NMR buffer (20 mM HEPES pH 7.0, 75 mM NaCl, 0.05% LMNG, 0.025% CHS, and 25 µM doxepin). Samples were concentrated to 280 μL in a Vivaspin-6 concentrator with a 30 kDa MWCO (Sartorius, Goettingen, Germany), 20 μL D_2_O was added and the sample was transferred to 5 mm Shigemi NMR tube.

^19^F-labeled H_1_R was prepared using the in-membrane chemical modification protocol [25]. Subsequent purification steps followed the above described protocol used for producing u-^15^N H_1_R.

### 2.5. Fluorescence Thermal Shift Assays

Fluorescence thermal shift assays were carried out as described previously [26]. H_1_R was extracted from *P. pastoris* membranes and purified in aqueous buffer containing LMNG/CHS mixed micelles without addition of any ligands. For each thermal shift assay sample, 5 µg of H_1_R was added to the buffer (50mM HEPES pH 7.0, 150mM NaCl, 0.05% DDM/CHS). The fluorescent CPM dye was added to each sample at a final concentration of 10 μM. Ligands were dissolved in a minimum amount of DMSO and added to each sample, which were then incubated in the dark on ice for 30 min and then transferred to quartz cuvettes. Experiments were carried out with a Cary Eclipse spectrofluorometer using quartz cuvettes (Starna Cells, Inc., Atascadero, CA, USA) over a linear temperature range from 20 °C to 90 °C heated at a constant rate of 2 °C/min. The excitation wavelength was 387 nm, and the emission wavelength was 463 nm. 

Thermal shift data were analyzed using the program Origin (OriginLab Corporation, Northampton, MA, USA). Raw data were fitted to a Boltzmann sigmoidal curve to determine the melting temperatures of each H_1_R sample.

### 2.6. NMR Spectroscopy

Two-dimensional [^1^H,^15^N] correlation spectra were recorded with a solution containing ~300 µM u-^15^N human H_1_R in 20 mM HEPES pH 7.0, 75 mM NaCl, 0.05% LMNG, 0.0025% CHS and 25µM doxepin. Two-dimensional [^15^N,^1^H]-transverse relaxation-optimized spectroscopy (TROSY) [27] correlation spectra were measured at 800 MHz ^1^H nutation frequency on a Bruker Avance III spectrometer running Topspin 3.6.2 and equipped with a 5-mm TXI cryoprobe. Experiments were measured at 42 °C. The sample temperature was calibrated using a standard sample (4% methanol in methanol-d4). The TROSY spectrum was recorded with acquisition periods of 80 ms in ^1^H and 16 ms in ^15^N, with a 1 s recycle delay for a total experimental time of about 22 h. NMR data were processed and analyzed in Topspin 3.5pl2 (Bruker Biospin, Billerica, MA, USA). Prior to Fourier transformation, the data matrices were zero filled to 1024 (t1) × 4096 (t2) complex points and multiplied by a Gaussian window function in the acquisition dimension and a 75°-shifted sine bell window function in the indirect dimension.

^19^F-NMR data were recorded with a solution containing 200 µM H_1_R in 20 mM HEPES pH 7.0, 75 mM NaCl, 0.05% LMNG, 0.0025% CHS and either 25 µM doxepin or 50 µM histamine. ^19^F-NMR experiments were measured at 600 MHz ^1^H nutation frequency on a Bruker Avance III HD spectrometer running Topspin 3.6.2 and equipped with a Bruker 5-mm BBFO probe. Experiments were measured at 7 °C, and the temperature was calibrated using a standard sample. ^19^F data were acquired with an acquisition period of 360 ms, 16k scans, and 0.3 s recycle delay for a total experimental time of about 3 h per experiment. NMR data were processed and analyzed in Topspin 3.5pl2 (Bruker Biospin, Billerica, MA, USA). Prior to Fourier transformation, the data were zero filled to 64k points and multiplied by an exponential window function with 30 Hz line broadening. NMR signals were referenced to an internal standard of trifluoro acetic acid (TFA), which has a chemical shift of −76.55 ppm relative to trichloro-fluoro-methane. 

## 3. Results

### 3.1. Design of an H_1_R Expression Vector for NMR Studies

**Figure 1:**
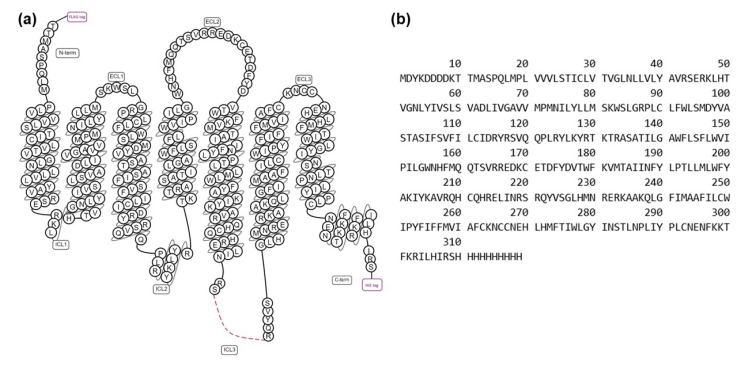
Design of a human H_1_R amino acid sequence for NMR studies. (**a**) Snake plot of the H_1_R receptor employed in the current study, as generated from the GPCRdb [31]. The red dashed line represents residues from the third intracellular loop that have been truncated from the native sequence. The “tag” labels at the N-terminus and C-terminus represent a FLAG tag and polyhistidine tag, respectively; (**b**) amino acid sequence of the human H_1_R employed in this study.

Construction of a human H_1_R expression vector for NMR studies was adapted from the design of the vector used to determine the crystal structure of the H_1_R complex with doxepin [9,28]. This H_1_R vector includes several modifications from the native receptor sequence: truncation of the first 19 amino acids, including two glycosylation sites, and replacement of the third intracellular loop (ICL3) with T4-lysozyme (T4L) to facilitate crystallization. This H_1_R vector expressed in *Pichia pastoris* was demonstrated to bind antagonists and agonists, including the endogenous ligand histamine, with affinities that were highly similar to native H_1_R expressed in insect cells and mammalian cells [9,28]. To preserve the ligand binding function, we retained all H_1_R endogenous amino acids present in the vector used for crystallization. For NMR studies, we removed the non-endogenous T4L, as the presence of T4L in ICL3 has been demonstrated to alter that native receptor function-related dynamics [29]. In our new vector, residues numbered 223^5.71^ to 229 and residues numbered 399 to 404^6.24^, comprising the intracellular ends of helices V and VI, respectively, were reinserted into the vector (superscripts indicate amino acid positions using the Ballosteros–Weinstein nomenclature [30]). The resulting amino acid sequence is shown in Figure 1. 

### 3.2. Optimization of H_1_R Expression and Purification Conditions

The H_1_R gene was placed into the open reading frame of a pPIC9K vector and transformed into electrocompetent *P. pastoris* Bg12 cells. Transformants containing a higher copy number of the H_1_R vector were screened in 4 mL cultures, and transformants showing higher expression of H_1_R relative to other transformants in a Western blot assay were selected for initial expression efforts. Expression of one of these transformants in standard buffered minimal glycerol (BMGY) and buffered minimal methanol (BMMY) media resulted in a yield of less than 0.25 mg of purified protein pure liter cell culture, as estimated from a Bradford assay.

**Figure 2:**
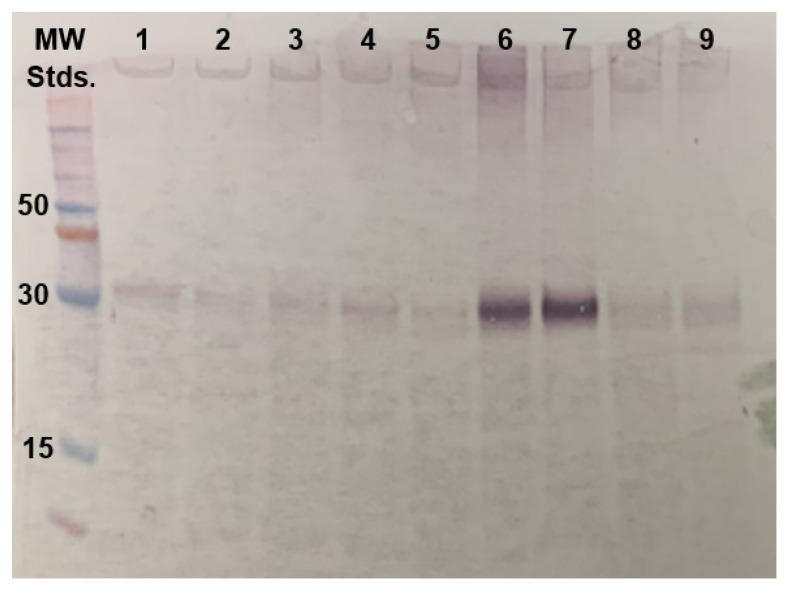
Optimization of H_1_R protein production from a Western blot analysis of protein expression conditions. Each lane represents a distinct set of experimental conditions used to express H_1_R in 4 mL cell culture (see Table 1). The intensity of a protein band appearing just above the 30 kDa molecular weight marker was used to identify optimal expression conditions. Cell culture conditions tested in lanes 6 and 7 were found to be optimal, and conditions in lane 6 were used for subsequent large-scale protein production.

To increase the yield of expressed H_1_R, multiple expression parameters were explored in 4 mL cell cultures, including induction temperature, duration of protein induction, addition of H_1_R ligands to the cell culture, and addition of DMSO to the cell culture. Three different temperatures of induction in BMMY media were explored: 22, 28, and 30 °C. Methanol feeding was varied from 24 up to 48 h. The addition of 1% (*w*/*v*) and 2% (*w*/*v*) of DMSO was also studied. The relative amounts of expressed proteins were analyzed via Western blot (Figure 2 and Table 1). 

Most of the tested parameters resulted in no to modest increases in the amount of expressed H_1_R. The addition of ligand showed the most pronounced effects. The presence of either 20 or 100 µM doxepin in the cell culture resulted in an approximate 10-fold increase in the yield of expressed H_1_R (Figure 2 and Table 1). The addition of 20 µM doxepin to BMMY media was thus implemented for all subsequent H_1_R production efforts. Production of H_1_R with the optimized expression conditions resulted in a yield of approximately 2 to 2.5 mg purified H_1_R per liter cell culture.

**Figure 3:**
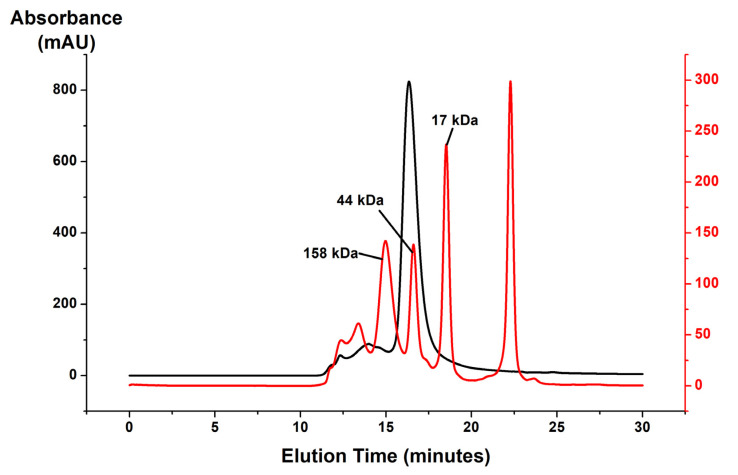
A representative analytical size exclusion chromatogram of purified H_1_R in solution containing 20 µM doxepin and LMNG/CHS mixed micelles is shown in black. Superimposed is a chromatogram of a solution containing molecular weight standards, shown in red. A mostly monodispersed population of purified receptor is observed for purified H_1_R, eluted at 16.3 min between the protein standards with molecular weights of 44 and 158 kDa.

H_1_R was isolated from *P. pastoris* membranes following a protocol adapted from the production and purification of human A_2A_AR from *P. pastoris* [20,22]. Purification of H_1_R in aqueous solutions containing doxepin and mixed micelles of either n-dodecyl-b-D-maltoside (DDM) and cholesterol hemisuccinate (CHS) or lauryl maltose neopentyl glycol (LMNG) and CHS resulted in a homogenous preparation. Analytical size exclusion chromatography of the purified H_1_R product showed a monodispersed peak with an estimated molecular weight above 50 kDa and below 150 kDa (Figure 3), which was similar to the observed elution times of samples of A_2A_AR prepared in LMNG/CHS mixed micelles. 

### 3.3. H_1_R Response to Ligand Binding

**Figure 4:**
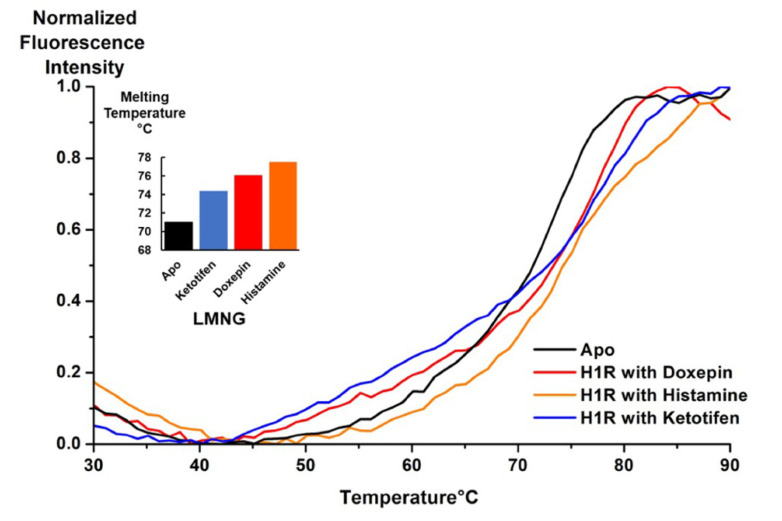
Fluorescence thermal shift assay curves are plotted for purified H_1_R in complex with several ligands and for a sample with no ligand added (apo). The experimental data were fitted to a Boltzmann sigmoidal function to calculate the unfolding temperatures (T_M_) of each sample, shown in the inset. Each experimental curve was normalized relative to the maximum observed fluorescence intensity.

To characterize the ability of purified H_1_R to bind ligands, we employed a fluorescence thermal shift assay using the thiol-specific fluorophore *N*-[4-(7-diethylamino-4-methyl-3-coumarinyl)phenyl]maleimide (CPM). This fluorophore interacts with cysteines present in the protein hydrophobic core as they become exposed to aqueous solvent due to thermal unfolding of the protein and has been used to characterize GPCR-ligand interactions [26]. To record the fluorescence thermal shift experiments, H_1_R was purified in an aqueous solution containing LMNG/CHS and without any ligand added. Several ligands were then individually mixed with aliquots of purified H_1_R and incubated for half an hour on ice prior to recording the temperature ramp experiments. To determine the melting temperature (T_M_) of each sample, the fluorescence data were fitted to a Boltzmann sigmoidal curve and the T_m_ was determined at the inflection point of the curve. All samples with ligands added showed moderate (3–4 °C) to more significant (8 °C) increases in the calculated T_M_ values over the sample with no ligand added (Figure 4), indicating that the purified protein recognized and could bind ligands. The T_M_ for H_1_R with no ligand added was determined to be 71.0 °C. The H_1_R sample with histamine added showed the highest determined T_m_ at 77.2 °C, and the curve showed a clear sigmoidal shape, consistent with cooperative unfolding.

### 3.4. Spectroscopic Characterization of Solutions Containing Purified H_1_R by 2-Dimensional TROSY NMR

**Figure 5:**
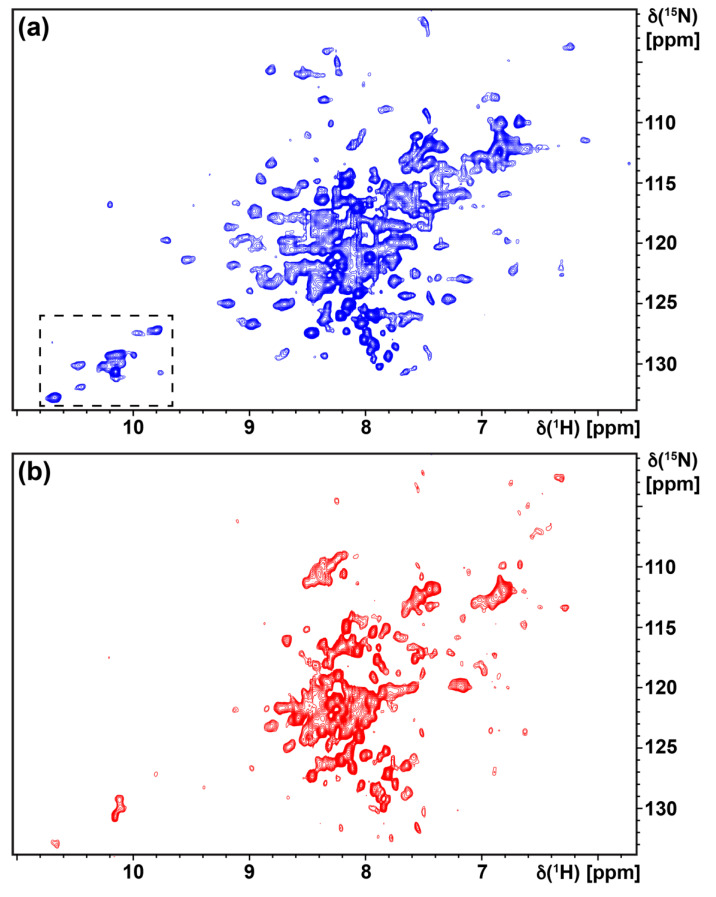
Two-dimensional [^15^N,^1^H]-TROSY correlation spectra are shown of [u-^15^N] H_1_R in complex with (**a**) the antagonist doxepin and (**b**) the agonist histamine. The dashed box in panel a represents the tryptophan indole region, which is shown on an expanded scale in Figure 6. Both spectra were recorded with ~300 µM H_1_R at a ^1^H NMR frequency of 800 MHz and experimental temperature of 42 °C for a total acquisition time of ~22 h per experiment.

Production of H_1_R for NMR studies followed the optimized expression and purification conditions described above. To assess the foldedness of purified H_1_R in solutions containing LMNG/CHS mixed micelles, we recorded [^15^N,^1^H]-TROSY two-dimensional spectra of u-^15^N H_1_R in complex with the ligands doxepin and histamine (Figure 5a,b). A TROSY spectrum of u-^15^N H_1_R in complex with doxepin recorded at 42 °C shows approximately 100 to 130 signals that are well dispersed from 6.5 to 10.5 ppm along the ^1^H chemical shift axis. About 20 to 25 signals of greater intensity are narrowly dispersed between 7.5 and 8.5 ppm along the ^1^H axis, which likely arise from more flexible loop regions of the protein. The approximately 25–30 well-resolved signals observed above 8.5 ppm are likely from amide groups in regular secondary structure and indicate that the protein is folded in conditions used for our NMR studies. A TROSY spectrum of u-^15^N H_1_R in complex with the agonist histamine shows fewer resolved signals than the complex with doxepin, reminiscent of earlier comparisons of TROSY spectra for antagonist and agonist complexes of human A_2A_AR [22]; however, a number signals are observed above 8.5 ppm and below 7.5 ppm, consistent with the thermal shift assays that indicate that the complex with histamine is folded. 

**Figure 6:**
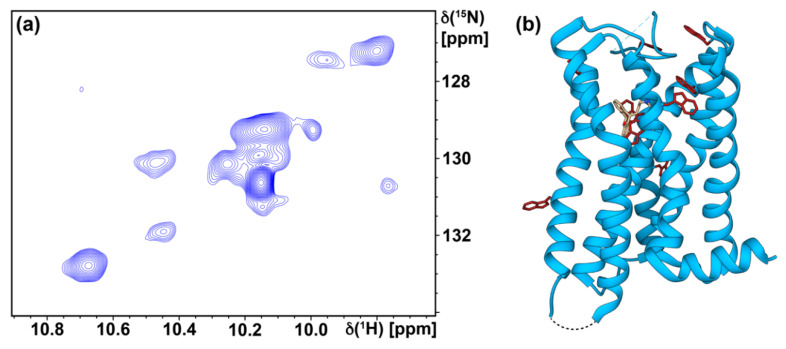
(**a**) An expanded view is shown of the tryptophan indole ^15^N–^1^H region of the TROSY correlation spectrum, corresponding to the dashed box in Figure 5a. (**b**) The crystal structure of the H_1_R complex with doxepin is shown in ribbon representation (PDB ID 3RZE [9]), and the 9 tryptophans in H_1_R are shown in red in stick representation. The blue dashed line represents missing electron density in the crystal structure for amino acids 168 to 174, and the black dotted line represents the location of T4L in the crystal structure. The bound doxepin ligand is shown in brown in stick representation.

**Figure 7:**
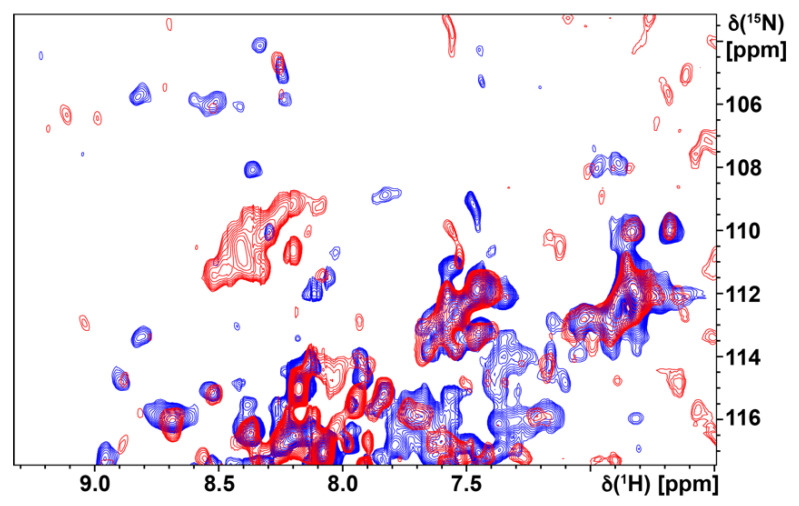
A superposition is presented of the TROSY spectra of u-^15^N H_1_R in complex with doxepin (blue) and histamine (red), showing an expanded view where signals from glycine and threonine ^1^H–^15^N amide signals are typically observed.

To provide further interpretation of the NMR data, we compared the TROSY spectrum with the crystal structure of the H_1_R complex with doxepin and more closely examined signals from the tryptophan indole ^15^N–^1^H region (Figure 5 and Figure 6a). There are four resolved signals of greater intensity in the tryptophan indole ^15^N–^1^H region of the TROSY spectrum and approximately five to six additional signals that are less intense but resolved. The crystal structure of the H_1_R complex with doxepin shows 9 tryptophans located in the transmembrane helices and extracellular loops (Figure 6b). Thus the number of observed signals in the Trp indole ^15^N–^1^H region of the TROSY spectrum is similar to the number of expected signals from all tryptophans. Comparing the spectra of the agonist and antagonist complexes shows some initial differences, for example in the region of the NMR spectra where glycine and threonine signals are typically observed (Figure 7). These initial spectra are highly encouraging considering that the protein is not deuterated, and thus we anticipate additional improvement in resolution upon incorporation of deuterium in the protein.

### 3.5. Response to Changes in Efficacy of Bound Drugs Monitored by ^19^F-NMR Spectroscopy

**Figure 8:**
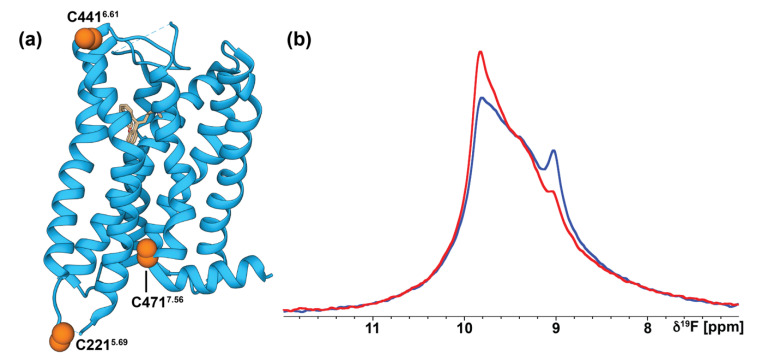
(**a**) The crystal structure of the H_1_R complex with doxepin is shown in ribbon representation (PDB ID 3RZE [9]). The 3 cysteines available for chemical modification are shown as orange spheres and labeled. (**b**) Superposition of ^19^F-NMR spectra of human H_1_R in complex with the antagonist doxepin (blue) and agonist histamine (red) recorded at 7 °C.

We assessed the potential for labeling with ^19^F-trifluoroethanethiol (TET) and whether we could detect a response to changes in the efficacy of bound ligands by preparing samples of H_1_R complexes with the antagonist doxepin and the endogenous agonist histamine. Based on analysis of the crystal structure of the H_1_R complex with doxepin, three endogenous cysteines are predicted to be more solvent accessible and thus available for ^19^F labeling via chemical modification with trifluoroethanethiol (TET). These cysteines are C221^5.69^, located at the intracellular end of helix V, cysteine 441^6.61^, located at the extracellular end of helix VI, and C471^7.56^, located at the intracellular end of helix VII (Figure 8a). While the TROSY experiments were recorded at 42 °C, the ^19^F NMR data were measured at 7 °C so that we could assess the data in the larger context of many previously reported ^19^F NMR GPCR studies [21,25,29,32,33]. Both ^19^F spectra of the complexes with doxepin and histamine show complicated line shapes containing more than three individual components (Figure 8b), suggesting the presence of multiple, simultaneously observed conformers for one or more of the labeled cysteines. Comparison of the ^19^F data between the complex with doxepin and the complex with histamine shows that individual components appear to have similar chemical shifts, for example the components near 9.0 and 9.8 ppm, with changes observed in the relative intensities of the individual components. This suggests that changes in relative populations of different conformers are potentially observed for complexes with different efficacies of bound ligands and that further ^19^F-NMR experiments with H_1_R variants containing single labeled cysteines may be used to characterize these different populations.

## 4. Discussion

### 4.1. Production of Stable-Isotope Labeled Human GPCRs in Pichia Pastoris

Production of human GPCRs for structure determination by crystallography or cryo-EM has been achieved mostly using insect cell expression [34], with some full-length class B and class F receptors expressed in mammalian cells. Though *P. pastoris* has been used to express a much smaller fraction of GPCRs for structure determination so far, the benefits of working with *P. pastoris* have motivated several studies of the potential to express functional GPCRs in this system. Surveys of the expression of about 30 different human GPCRs demonstrated that a sizeable fraction of functional receptors could be expressed at milligram to multi-milligram scales [13,35]. Systematic evaluation of expression conditions also demonstrated that yields of more poorly expressed receptors could be significantly improved with optimized protocols [11].

*P. pastoris* offers multiple advantages for the expression of human membrane proteins for NMR studies, which include the ability to produce functional proteins with high levels of deuterium incorporation [36]. A growing number of membrane proteins have been expressed in *P. pastoris* for NMR experiments in both aqueous solutions and solid state samples, including human aquaporin 1 [37], human aquaporin 2 [38], and fungal rhodopsin from *Leptosphaeria maculans* [39]. Initial NMR data with the human GPCRs CB1 and OX2 also appear to be promising [24]. The NMR data presented here demonstrate that functional human H_1_R can be produced at sufficient quantities for NMR studies and establish an important step in the application of NMR to study the energy landscape of additional human GPCRs.

### 4.2. Tryptophans as NMR Probes of GPCR Structure-Function Relationships

Both endogenous and extrinsically introduced tryptohans are sensitive reporters of function-related changes in the GPCR structure, particularly when nearby other aromatic amino acids that create significant ring current shifts [40]. Further, tryptophan indole ^15^N–^1^H signals are typically well-separated from the majority of amide signals in [^15^N,^1^H] heteronuclear two-dimensional experiments, which facilitates their assignment by amino acid replacement. We explored the potential use of tryptophans in future NMR studies of H_1_R structure–function relationships by calculating the ring current shifts for H_1_R tryptophan indole ^15^N–^1^H signals from the crystal structure of the H_1_R complex with doxepin using the program Molmol [41]. The calculated ring current shifts show a relatively narrow range spanning approximately 0.3 ppm, with the notable exception of Trp 428^6.48^ (Table 2; superscripted text denotes the Ballosteros–Weinstein nomenclature used to report GPCR amino acid positions relative to a consensus sequence [30]). The calculated ring current shift for Trp 428^6.48^ shows a pronounced shift of −0.56 and −0.78 ppm for the Hε1 and Nε1 chemical shifts, respectively. Comparably large ring current shifts were also observed for the corresponding Trp246^6.48^ indole ^15^N–^1^H lines in TROSY NMR spectra of human A_2A_AR [19,22]. The single largest contribution to these ring current shifts was the nearby Phe242^6.44^ of the highly conserved P-I-F activation motif [19,22], and the chemical shifts of Trp246^6.48^ were found to be highly sensitive to the efficacy of bound drugs. Thus, we anticipate that Trp 428^6.48^ in H_1_R will be a sensitive probe of structure–function relationships.

## 5. Conclusions

We present a protocol for the expression and purification of the human H_1_ histamine receptor in *Pichia pastoris*, which can be applied to economically produce stable-isotope labeled GPCR samples for NMR experiments. Evaluation of protein production parameters revealed that addition of ligand during protein expression resulted in a ten-fold increase in the yield of expressed receptor. Purification of expressed H_1_R in buffer containing either DDM/CHS mixed micelles or LMNG/CHS mixed micelles yielded a monodispersed and homogeneous preparation. Purified H_1_R responded to the addition of known ligands, as monitored by fluorescence thermal shift assays, with the addition of the ligand histamine resulting in the largest increase in the protein melting temperature. Two-dimensional [^15^N,^1^H]-TROSY NMR spectra of u-^15^N H_1_R in buffer containing LMNG/CHS mixed micelles show well-dispersed signals consistent with a folded protein and comparison of TROSY spectra of H_1_R complexes with an antagonist and an agonist show changes in the distribution of signals. Evaluation of the tryptophan indole ^15^N–^1^H region of the TROSY correlation spectrum of the antagonist complex shows the presence of a similar number of signals as expected from the crystal structure of H_1_R. ^19^F-NMR spectra of H_1_R show differences related to changes in the efficacy of bound drugs. Expression of stable-isotope labeled human receptors is a bottleneck in the application of NMR spectroscopy to map the energy landscapes for most human GPCRs. While *P. pastoris* is a promising host for GPCR expression, only a few receptors have been expressed successfully in *P. pastoris* for NMR studies. This work demonstrates an additional example of a human GPCR that can be prepared from *P. pastoris* for NMR experiments.

## Figures and Tables

**Table 1:** Parameters used for testing H_1_R expression conditions corresponding to the Western blot analysis in Figure 2.

Lane.	Induction Temperature	Methanol Induction Time	Concentration of Doxepin Added	DMSO% (*w*/*v*)
1	28 °C	36 h	0	0
2	22 °C	36 h	0	0
3	30 °C	36 h	0	0
4	28 °C	24 h	0	0
5	28 °C	48 h	0	0
6	28 °C	36 h	20 µM	0
7	28 °C	36 h	100 µM	0
8	28 °C	36 h	0	1%
9	28 °C	36 h	0	2%

**Table 2:** Calculated tryptophan indole ^15^N and ^1^H ring current shifts using the structure of H_1_R in complex with doxepin (PDB ID 3RZE). Calculated ring current shifts for the conserved “toggle switch” tryptophan, Trp^6.48^, are highlighted in the grey row.

Trp Position	B-W Notation ^1^	Δδ_RC_ [ppm] Hε1	Δδ_RC_ [ppm] Nε1
93	ECL1	0.03	0.02
103	3.28	−0.15	−0.12
152	4.50	0.09	0.11
158	4.56	−0.17	−0.11
165	ECL2	0.05	0.05
189	5.37	0.01	0.03
208	5.56	0.12	0.15
428	6.48	−0.57	−0.78
455	7.40	0.11	0.10

^1^ Ballosteros–Weinstein nomenclature.
